# Catheterization of the petrosal sinus: possible advantage of desmopressin stimulation in Cushing disease

**DOI:** 10.20945/2359-4292-2026-0064

**Published:** 2026-05-01

**Authors:** Heraldo Mendes Garmes, Sara Calazans de Siqueira, Karla Borges Daniel, Marilia Bortolotto Felippe Trentin, Mariana Tazima Fujiwara, Caroline Schinoll, Mateus Dal Fabbro, Fabio Rogerio, Leonardo Deus-Silva, Fabiano Reis

**Affiliations:** 1 Serviço de Endocrinologia, Departamento de Medicina Clínica, Faculdade de Ciências Médicas, Universidade Estadual de Campinas (Unicamp), Campinas, SP, Brasil; 2 Departamento de Neurocirurgia, Faculdade de Ciências Médicas, Universidade Estadual de Campinas (Unicamp), Campinas, SP, Brasil; 3 Departamento de Patologia, Faculdade de Ciências Médicas, Universidade Estadual de Campinas (Unicamp), Campinas, SP, Brasil; 4 Setor de Neurorradiologia Intervencionista; Departamento de Radiologia, Faculdade de Ciências Médicas, Universidade Estadual de Campinas (Unicamp), Campinas, SP, Brasil; 5 Departamento de Radiologia e Oncologia, Faculdade de Ciências Médicas, Universidade Estadual de Campinas (Unicamp), Campinas, SP, Brasil

**Keywords:** Cyclic hypercortisolism, desmopressin, petrosal sinus, Cushing’s syndrome

## Abstract

Thediagnosis of Cushing syndrome can be challenging, particularly when bilateral inferior petrosal sinus sampling (BIPSS) is required to differentiate ectopic tumors from pituitary adrenocorticotropic hormone (ACTH)-releasing tumors. When corticotropin-releasing hormone (CRH) is used as a stimulus, a state of hypercortisolism is required to suppress CRH receptors in pituitary corticotrophs, preventing the release of ACTH by normal pituitary tissue. By contrast, desmopressin acts through vasopressin receptors that are preferentially expressed in ACTH-secreting pituitary tumors, suggesting potentially different physiological behaviors during stimulation. We present the case of a 26-year-old woman with ACTH-dependent Cushing’s syndrome who underwent desmopressin-stimulated BIPSS. Hormone levels on the day of catheterization indicated eucortisolism; however, ACTH levels in the petrosal sinus confirmed the diagnosis of Cushing disease. Following transsphenoidal surgery, immunohistochemical analysis confirmed a corticotroph adenoma with positive Tpit and ACTH staining. We hypothesize that desmopressin stimulation during BIPSS may provide diagnostic information even in the absence of biochemical confirmation of hypercortisolism at the time of the procedure, because vasopressin receptors are expressed in pituitary corticotroph adenomas rather than in normal corticotroph cells. Further studies are needed to evaluate and confirm this hypothesis.

## INTRODUCTION

In patients with a confirmed diagnosis of ACTH-dependent Cushing syndrome, intravenous desmopressin testing is not a reliable method for differentiating pituitary tumors from ectopic ACTH-producing tumors, as a subset of ectopic tumors express aberrant amounts of vasopressin receptors and respond to desmopressin stimulation, resulting in increased ACTH levels in the peripheral circulation ^(1)^.

Bilateral inferior petrosal sinus sampling (BIPSS) with CRH stimulation is the gold standard test used for this differential diagnosis, but CRH is currently unavailable. Furthermore, in patients with cyclical Cushing syndrome, catheterization of the inferior petrosal sinus should be performed during hypercortisolemic phases to prevent normal corticotroph cells from responding to CRH stimulation ^(2)^. Desmopressin has been successfully used in BIPSS and is currently considered equally accurate as CRH because ACTH-producing pituitary tumors overexpress vasopressin receptors ^(3,4)^.

Scheduling BIPSS with CRH stimulation during hypercortisolemic phases can be challenging. This case illustrates the use of desmopressin as an alternative to CRH in BIPSS and highlights the discussion of its physiological basis, given that normal pituitary tissue does not express vasopressin V2 receptors.

## CASE REPORT

A 26-year-old previously healthy woman was referred to our neuroendocrinology specialized center 6 months after the beginning of the investigation for weight gain, violaceous striae on the abdomen, fatigue, acne, myalgia, lower limb edema, moon facies, hair loss, and hirsutism. Initial laboratory investigations revealed intermittent ACTH-dependent hypercortisolism, followed by a period of normal urinary free cortisol (UFC) levels (**Table 1**).

**Table 1:** Results showing ACTH-dependent intermittent hypercortisolism

Exams 2023	April	May	June	October	November BIPSS
Cortisol	35.7		47.0	6.3	6.31
ACTH	198.0		156.0		
1 mg-DST	28.16	20.3			
UFC 24h				19 (13-85)	51 (36-137)

ACTH: adrenocorticotropic hormone in pg/mL; 1 mg-DST: 1 mg-dexamethasone suppression test; UFC: urinary free cortisol in ug/dL.

**Figure 1:**
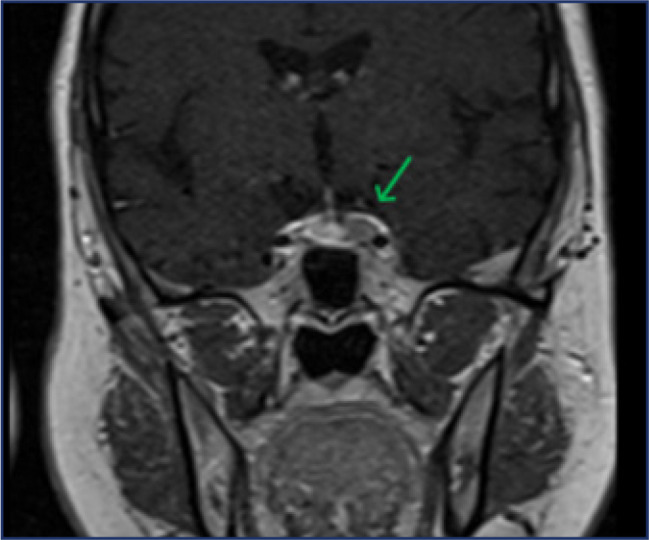
Magnetic resonance imaging of the sella turcica: Coronal T1-weighted image shows a left pituitary microadenoma.

Pituitary magnetic resonance imaging (MRI) revealed an irregular nodule located on the lateral aspect of the adenohypophysis, projecting into the left cavernous sinus and in contact with the internal carotid artery, measuring 0.9 × 0.9 × 0.6 cm (**Figure 1**).

The patient underwent BIPSS with desmopressin (10 µg), and correct catheterization was verified by angiography and contrast infusion.

At the time of the procedure, eucortisolism was confirmed by a basal serum cortisol concentration of 6.31 µg/dL and a 24-hour UFC level of 51 µg/24 h collec-ted the day before (reference range: 36-137 µg/24 h). Based on the current evidence, if CRH had been used as the stimulus, the procedure would have been postponed because of normal cortisolemia.

In the BIPSS test performed at our institution, peripheral blood sampling is extended to 30 minutes after desmopressin infusion to assess the peripheral ACTH response, as in an acute desmopressin test. The procedure involves administering desmopressin through a vein in one forearm while blood is collected from the contralateral arm to differentiate ACTH-dependent Cushing syndrome from nontumoral endogenous hypercortisolism (pseudo-Cushing syndrome).

The peripheral serum cortisol concentration after the desmopressin stimulus ranged from 6.42 mcg/dL at baseline to 19.11 mcg/dL, an increase of 198%, while the basal ACTH concentration ranged from 83.14 pg/mL to 149.40 pg/ml, an increase of 79%. These results from peripheral samples favored the diagnosis of ACTH-dependent Cushing syndrome rather than nontumorous hypercortisolism. Still, they did not exclude the possibility of an ectopic ACTH-producing tumor that may express vasopressin receptors. In the inferior petrosal sinus samples, the high central-to-peripheral ACTH gradient confirmed the presence of vasopressin receptors in a pituitary ACTH-secreting tumor, confirming the diagnosis of cyclic CD (**Table 2**).

**Table 2:** Results of samples collected from the inferior petrosal sinuses and peripheral veins

BIPSS	Basal	1 minute	3 minutes	5 minutes	10 minutes	30 minutes
ACTH IPS L	756.2	1155.0	> 1500.0	1454.0	> 1500.0	
ACTH IPS R	316.0	387.0	368.0	1095.0	> 1500.0	
ACTH P	83.1	58.8	73.0	77.5	121.7	149.4
PRL IPS L	86.1					
PRL IPS R	61.0					
PRL P	13.9					
Cortisol P	6.4				8.9	19.1
IPS L/P	9.1	19.6	20.5	18.7	0.9	
IPS R/P	3.1	6.6	5.0	14.1	12.3	

ACTH: adrenocorticotropic hormone in pg/mL; IPS: inferior petrosal sinus; L: left; R: right; P: peripheral; PRL: prolactin in ng/mL.

The patient underwent transsphenoidal resection of the microadenoma. Immunohistochemical analysis confirmed a corticotroph tumor with 80%-90% ACTH positivity and 80% Tpit positivity in neoplastic cells. She experienced clinical and biochemical remission after surgery.

The most recent postoperative follow-up tests revealed a 24-hour UFC of 23 µg/24 h (reference range: 13-85), a late-night salivary cortisol concentration of 1.5 nmol/L (23:00 h; reference value < 7.6), and a serum cortisol concentration of 0.7 µg/dL after dexamethasone suppression (reference value < 1,8).

## DISCUSSION

This case illustrates that, in ACTH-dependent Cushing syndrome, BIPSS with desmopressin stimulation may help differentiate a pituitary from an ectopic ACTH source, even during the eucortisolemic phases of cyclic Cushing syndrome.

First, it is worth noting that, because our patient presented with a normal urinary free cortisol level on the day of the BIPSS, the possibility of spontaneous remission of CD could be considered. A recent systematic review of the literature evaluating cases of spontaneous remission in Cushing disease revealed that tumor apoplexy is considered the most likely etiology for this rare phenomenon ^(5)^; however, our patient did not present clinical signs or radiological evidence of pituitary apoplexy. Furthermore, the greatly elevated ACTH levels in the petrosal sinuses before and after desmopressin stimulation indicate the presence of an ACTH-producing pituitary tumor, which was found intraoperatively and confirmed histopathologically with immunohistochemistry demonstrating ACTH positivity in 80%-90% and T-Pit positivity in 80% of the neoplastic cells, making spontaneous remission an unlikely explanation for this patient.

A recent meta-analysis confirmed that performing BIPSS using desmopressin as a stimulus for ACTH secretion yields results comparable to those obtained with CRH stimulation for determining the etiology of ACTH-dependent Cushing syndrome ^(3)^.

Regarding the literature on cyclic Cushing syndrome, cases of misdiagnosis due to BIPSS with CRH stimulation performed during periods without hypercortisolemia are common ^(6-8)^, sometimes falsely suggesting EAS in patients with cyclic CD ^(7)^ or CD in patients with EAS ^(6,7)^. Notably, these cases were later confirmed histologically. Albani and cols. ^(6)^ reported the case of a patient with cyclic EAS who, after undergoing an initial BIPSS during the eucortisolemic phase, underwent unnecessary pituitary surgery. The true EAS diagnosis was confirmed only after a second BIPSS was performed during the active phase of the tumor.

According to a recent review by Nowak and cols., the positive and negative predictive values of BIPSS with CRH decrease from 100% to 42% and from 100% to 73%, respectively, when performed outside hypercortisolemic phases ^(9)^. Therefore, BIPSS with CRH stimulation should be performed only during an active disease period. Additionally, notably, a sustained period of hypercortisolism is necessary to suppress normal corticotroph function, which requires serial cortisol assessments before BIPSS with CRH stimulation is performed in patients with cyclic ACTH-dependent CS. This requirement for hypercortisolism represents a major challenge, significantly delaying diagnosis and treatment ^(10)^.

Furthermore, some authors have reported that the prevalence of cyclic Cushing syndrome is substantial, ranging from 14% to 19% of patients with CD, suggesting that this condition is not as rare as previously believed ^(10-13)^.

Desmopressin is a vasopressin analog that is relatively selective for V2 renal receptors but has a weak affinity for V3 pituitary receptors in healthy individuals ^(14)^. However, due to the overexpression of vasopressin receptors in corticotroph adenomas and some ectopic ACTH-secreting tumors, desmopressin induces a rapid increase in ACTH release in affected patients. By contrast, studies have shown that this increase is absent in normal individuals or those with non-neoplastic hypercortisolism ^(15,16)^.

With respect to the need for hypercortisolism when performing BIPSS to investigate the source of ACTH in ACTH-dependent Cushing syndrome, two distinct pathophysiological scenarios can be considered: When corticotropin-releasing hormone (CRH) is used as the stimulus, the ACTH response depends on the expression of CRH receptors, which are present in both pituitary corticotroph adenomas and normal pituitary corticotroph cells. In this setting, sustained hypercortisolism is required to suppress normal corticotroph responsiveness; in its absence, CRH-induced ACTH secretion from normal corticotrophs may occur, potentially leading to false-positive results and a misdiagnosis of Cushing disease ^(17)^. By contrast, when desmopressin is used as the stimulus, the ACTH response is primarily determined by the expression of vasopressin receptors, which are characteristically overexpressed in ACTH-secreting pituitary adenomas but minimally or not expressed in normal corticotroph cells. Based on this physiological distinction, the lack of suppression of CRH receptors in normal corticotrophs by hypercortisolism is less relevant for desmopressin-stimulated BIPSS, potentially allowing discrimination between pituitary and ectopic ACTH secretion even during periods of normocortisolism.

Supporting this concept, studies using desmopressin stimulation on peripheral blood samples have differentiated patients with CD from those with pseudo-Cushing states, demonstrating that ACTH-producing pituitary tumors respond to desmopressin, whereas the normal pituitary gland does not ^(16)^.

**Figure 2:**
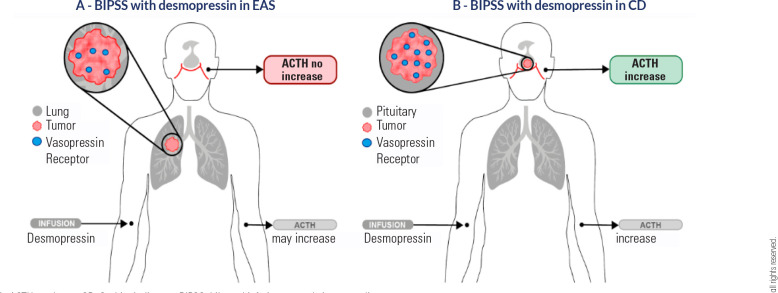
Results of ACTH measurement after desmopressin infusion in patients with EAS (**A**) or CD (**B**). Because pituitary tumors are known to express vasopressin receptors, increased ACTH in the petrosal sinus occurs only in patients with CD, regardless of cortisolemia.

EAS: ectopic ACTH syndrome; CD: Cushing’s disease; BIPSS: bilateral inferior petrosal sinus sampling.

Based on this observation, we question whether desmopressin-stimulated BIPSS could have diagnostic utility even when performed during the eucortisolemic phase. In this context, an increased central-to-peripheral ACTH gradient would support a pituitary source of ACTH secretion, which is consistent with the presence of a corticotroph adenoma expressing vasopressin receptors. Conversely, in ectopic ACTH-producing tumors, desmopressin stimulation would not be expected to generate a significant central-to-peripheral ACTH gradient (**Figure 2**).

Hypercortisolism remains the standard prerequisite for BIPSS, and our findings should be interpreted as an exploratory observation that provides a basis for further evaluation by the scientific community. Thus, further studies including patients with cyclic Cushing disease during periods of eucortisolism are needed to determine whether these results can be reproduced and whether all these patients will respond to vasopressin stimulation in the BIPSS.

## CONCLUSION

Our case report suggests that BIPSS with desmopressin stimulation might be used for the etiological diagnosis of ACTH-dependent cyclic Cushing syndrome, even during the normocortisolemic phase. This strategy may facilitate earlier diagnosis and treatment, mitigating the challenges posed by the requirement for hypercortisolism in conventional BIPSS with CRH stimulation. However, further studies are needed to confirm these findings and clarify their clinical applicability.

## Data Availability

the original contributions presented in the study are included in the article. Further inquiries can be directed to the corresponding author.
